# Poster Presentations

**DOI:** 10.1080/13577140120097067

**Published:** 2001-11


### POSTER PRESENTATIONS

### SURGERY

### 015 Risk of Amputation following Limb Salvage Surgery with Endoprosthetic Replacement

Lee M Jeys, Robert J Grimer, Simon R Carter, Roger M Tillman
*Royal Orthopaedic Hospital Oncology Service, Royal Orthopaedic Hospital*

*Objective:* Endoprosthetic Replacements (EPRs) are one of the most commonly used types of limb salvage following surgical excision of bone tumours. The advantage of Endoprosthetic Replacements are their initial reliability and the rapid restoration of function along with their ready availability. The problems with Endoprosthetic Replacements are the long term problems of wear, loosening, infection and mechanical failure. Increasing and insolvable problems may lead to the necessity for amputation. This paper assesses the risk of amputation following Endoprosthetic Replacement.
*Methods:* Information was collected from a prospectively recorded database in the unit. Information was used to identify the patients having undergone EPRs and those with subsequent amputations.
*Results:* A total of 1262 patients have undergone Endoprosthetic Replacement surgery at our centre in the past 34 years. They have a total of 6507 patient years of follow up. A total of 112 patients have had subsequent amputation (8.9%). The reasons for amputation were local recurrence in 71(64.4%), infection in 38(33.9%), mechanical failure in 2(1.8%) and continued pain in 1 case(0.8%). The risk of amputation was greatest in the proximal tibia 15.5% (n=38/246), followed by pelvis 10.2%(5/49), and femur 7.4% (n=58/784), whilst the risk of amputation was least in the humerus at 6.4% (n=11/182). The time to amputation varied from 2 days to 16.37 years, with a mean of 31 months. The risk of amputation decreased with time although 10% of the amputations took place more than 5 years after implantation.
*Conclusion:* The greatest risk of amputation is in the first 5 years and is due to local recurrence, whilst infection poses the next greatest threat. The risk decreases with time. Attempts to control both local recurrence and infection will decrease the need for amputation. Late failure of the EPRs, even in young patients does not seem to be a major cause of amputation thus far.

### 017 Reconstruction following Total Scapular Resection: Analysis of Humeral Suspension Versus Endoprosthetic Reconstruction

Jacob Bickels[1], James C Wittig[2], Yehuda Kollender[1], Kristen L. Kellar-Graney[2], Isaac Meller[1], Martin Miles Malawer[2]
[1]*National Unit of Orthopedic Oncology Tel-Aviv Sourasky Medical Center,* [2]*Division of Orthopedic Oncology Washington Cancer Institute Washington Hospital Center*

*Objective:* Total scapular resection causes a significant functional loss because of the sacrifice of the glenoid, which serves as a stable base for shoulder motion. The authors analyze their experience with two types of reconstructions following total scapular resection; suspension of the humeral head from the clavicle without endoprosthetic reconstruction of the scapula and endoprosthetic scapular reconstruction.
*Methods:* Between 1979 and 1997, the authors treated 23 patients with scapular tumors that required total scapular resection. Patients were diagnosed with 14 bone and 9 soft–tissue tumors. Resection included total scapulectomy in 12 patients and enbloc resection of the scapula and humeral head in 11 patients. Reconstruction: All eleven patients who had resection of their humeral head underwent reconstruction of the humerus with endoprosthesis. Scapular endoprosthesis was further installed in 7 patients and suspension of the humeral head from the clavicle with a Dacron tape was performed in 16 patients (Suspension of the prosthetic humeral head from the clavicle – 4 patients; suspension of the native humeral head from the clavicle – 12 patients). Endoprosthetic reconstruction of the scapula was feasible only when the periscapular musculature was sufficient for endoprosthetic attachment and coverage. The scapular prosthesis was attached to the prosthetic humeral head with a Goretex® sleeve, which served as an artificial joint capsule. All patients were followed for a minimum of 2 years; follow-up included physical examination, radiological evaluation and functional evaluation according to the American Musculoskeletal Tumor Society system.
*Results:* Elbow range-of-motion and hand dexterity were similar in the two groups of patients. However, compared with patients who had undergone humeral suspension, those who had scapular endoprosthesis had better abduction (60°-90° vs. 10°-20°) of the shoulder joint. Moreover, these patients had better cosmetic appearance of the shoulder girdle. There were no deep wound infections, prosthetic failures, or secondary amputations. Overall, 6 patients who had scapular prosthesis (86%) and 10 patients who had humeral suspension (62%) had a good-to-excellent functional outcome.
*Conclusion:* The number of patients who underwent a scapular endoprosthetic reconstruction is small and does not allow a valid statistical analysis; however, the authors feel that scapular endoprosthesis reconstruction is associated with better functional and cosmetic outcomes, when compared to humeral suspension. The authors recommend reconstruction of the scapula with endoprosthesis when periscapular musculature, remaining after tumor resection allows attachment and coverage of the prosthesis.

### 018 Extensile Exposure of the Axilla

Eran Maman[1], Jacob Bickels[1], James C Wittig[2], Kristen L. Kellar-Graney[2], Yehuda Kollender[1], Isaac Meller[1], Martin Miles Malawer[2]
[1]*National Unit of Orthopedic Oncology Tel-Aviv Sourasky Medical Center,* [2]*Division of Orthopedic Oncology Washington Cancer Institute Washington Hospital Center*

*Objective:* Tumors of the axilla impose a surgical difficulty because they are usually large at presentation and in close proximity to the major neurovascular bundle of the upper extremity. Attempted tumor resection via the base of the axilla is difficult because of limitations in full visualization of the lesion and identification and mobilization of the neurovascular bundle prior to resection. The authors have used a safe and reliable exposure for these situations.
*Methods:* Between 1980 and 1997, 35 patients underwent extensile exposure of an axillary tumor. Diagnoses included 19 primary and 16 metastatic tumors of the axilla. The axillary cavity was fully exposed via the deltopectoral groove after detachment and reflection of two layers of muscles: first, the pectoralis major and, second, the coracoid origin of the pectoralis minor, coracobrachialis, and the short head of the biceps muscle. This surgical approach allowed full tumor visualization and determination of the exact anatomic relation of the tumor to the neurovascular bundle and as a result, tumor resectability. Following resection, the pectoralis minor and conjoined tendons were reattached to the coracoid process with a nonabsorbable suture, and the pectoralis major was reattached to its insertion site on the proximal humerus in the same manner.

DOI: 10.1080/13577140120097067

*Results:* Exposure revealed a safe plane of dissection between the tumor and the major neurovascular bundle in 23 patients and invasion of the major neurovascular bundle in 12 patients who subsequently underwent a forequarter amputation. At the most recent follow-up, none of these patients had functional limitation, which could be attributed to the extensile approach itself. All patients gained their presurgical pectoralis major and biceps function. Complications in the group of patients that underwent tumor resection included three (13%) superficial wound infections. Due to intended, enbloc resection of an involved nerve with the tumor, two nerve palsies (8.7%) were documented. None of the remaining 21 patients had numbness, paresthesias, or nerve pain. There were three (13%) local recurrences; two were managed with wide excision and adjuvant radiation therapy and one necessitated amputation.
*Conclusion:* The extensile exposure of the axilla allows full visualization of axillary tumors. It allows determination of tumor resectability and safe and reliable resection, when indicated. This exposure is associated with good functional outcome and an acceptable morbidity. The extensile axillary exposure is recommended in the management of axillary tumors.

**019 Enchondroma of the Hand: Management with Curettage and Cemented Intramedullary Hardware**

Jacob Bickels[1], James C. Wittig[2], Yehuda Kollender[1], Kari L Mansour[2], Isaac Meller[1], Martin M Malawer[2]
*[1]Tel-Aviv Sourasky Medical Center National Unit of Orthopedic Oncology, [2]Division of Orthopedic Oncology Washington Cancer Institute Washington Hospital Center*

*Objective:* Surgical removal by means of curettage is the mainstay of treatment of enchondromas of the hand. Methods of reconstruction after tumor removal usually entail no reconstruction or filling of the tumor cavity with a bone graft. These techniques necessitate a prolonged period of protected activity until bone healing of the tumor cavity occurs. The authors have utilized hardware and bone cement for the purpose of reconstruction of the tumor cavity. This technique provides immediate mechanical stability and allows early mobilization.
*Methods:* Between 1986 and 1999 the authors treated 13 patients (8 females, 4 males) who ranged in age from 23 to 58 years (median, 32 years) and diagnosed with enchondroma of the hand. Eight patients presented with a pathological fracture. Anatomic locations included: metacarpal bones – 5, proximal phalanx – 4, and middle phalanx – 4. Tumors were approached through the retained thinned or destroyed cortex to minimize additional bone loss. Surgery included removal of all gross tumors with hand curettes; this was followed by high-speed burr drilling of the inner reactive bone shell. Reconstruction included intramedullary metal wire along the longitudinal axis of the cavity and polymehylmethacrylate (PMMA). Full activity as tolerated was allowed immediately after surgery. All patients were followed for more than 2 years; Follow-up included physical and radiological evaluation and functional evaluation.
*Results:* Following surgery, all patients returned to their presurgical functional capability within two weeks. At the last follow-up, none of the patients had local tumor recurrence and although three patients had 15° to 20° decrease in flexion of the metacarpophalangeal joint, none reported a functional limitation. There were no postoperative infections or fractures.
*Conclusion:* Reconstruction of the tumor cavity, remaining after curettage of enchondroma of the hand, with intramedullary hardware and PMMA provides immediate mechanical stability and allows early mobilization. This technique is associated with good short- and long-term functional outcomes.

**020 Distal Femur Resection with Endoprosthetic Reconstruction: A Long Term Follow-Up Study**

Jacob Bickels[1], James C Wittig[2], Yehuda Kollender[1], Robert M Henshaw[2], Robert S Neff[2], Isaac Meller[1], Martin M Malawer[2]
*[1]National Unit of Orthopedic Oncology Tel-Aviv Sourasky Medical Center, [2]Division of Orthopedic Oncology Washington Cancer Institute Washington Hospital Center*

*Objective:* The distal femur is a common site for primary and metastatic bone tumors. It is, therefore, a frequent site in which to perform a limb-sparing surgery. The authors describe their experience with distal femur resection and endoprosthetic reconstruction
*Methods:* There were 61 males and 49 females who ranged in age from 10 to 80 years. Nineteen patients were younger than 12 years of age. Diagnoses included 99 malignant tumors of bone, 9 benign-aggressive lesions, and 2 non-neoplastic conditions that had caused massive bone loss or articular surface destruction. Endoprosthetic reconstruction included 73 modular, 27 custom-made, and 10 expandable prostheses. Only eight patients had a constrained knee mechanism; the remaining patients underwent reconstruction with a rotating-hinge knee mechanism. All prostheses were fixed with bone cement. Soft-tissue reconstruction included 21 medial, 3 lateral, and one bilateral gastrocnemius flaps. All patients were followed for more than 2 years (range, 2-16.5 years; average, 5.2 years); Follow-up included physical and radiological evaluation and functional evaluation according to the American Musculoskeletal Tumor Society System.
*Results:* Function was estimated to be good or excellent in 94 patients (85.4%), moderate in 9 patients (8.2%), and poor in 7 patients (6.4%). Patients who underwent reconstruction with a rotating-hinge knee mechanism were more likely to have a good-to-excellent functional outcome (91%) than those who underwent reconstruction with a constrained knee mechanism (50%). Complications included six deep wound infections (5.4%), which resulted in three amputations, two prosthetic revisions, and one wound debridement. There were six local recurrences, five of which were treated with wide local excision, and one necessitated amputation. Overall, there were 14 revision surgeries; these included replacement of failed polyethylene component in 6 patients and prosthetic revision in 8 patients (aseptic loosening – 5; deep infection – 2; and radiation bone necrosis –1). There were six local recurrences, five of which were treated with wide local excision, and one necessitated amputation. Prosthetic survival was 94% at 5 years and 91% at 10 years and overall limb salvage rate was, therefore, 96%.
*Conclusion:* Distal femur resection with endoprosthetic reconstruction is a safe and reliable procedure, which provides good local tumor control. The use of cemented endoprostheses, combined with a rotating-hinge knee mechanism provide immediate mechanical stability, allows early mobilization and good-to-excellent function in most patients. The use of endoprostheses is recommended by the authors for reconstruction of large segmental defects of the distal femur. Distal femur resection with endoprosthetic reconstruction is a safe and reliable procedure, which provides good local tumor control. The use of cemented endoprostheses, combined with a rotating-hinge knee mechanism provide immediate mechanical stability, allows early mobilization and good-to-excellent function in most patients. The use of endoprostheses is recommended by the authors for reconstruction of large segmental defects of the distal femur.

**021 Constrained (Rotator Cuff Substituting) Total Scapula Prosthesis: "Anatomic" Approach for Reconstructing the Shoulder Girdle following High-Grade Sarcoma Resection**

James C Wittig[1], Jacob Bickels[2], Felasfa Wodajo[1], Kristen L. Kellar-Graney[1], Martin M Malawer[1]
*[1]Division of Orthopedic Oncology Washington Cancer Institute Washington Hospital Center, [2]National Unit of Orthopedic Oncology Tel-Aviv Sourasky Medical Center*

*Objective:* High-grade sarcomas arising from the scapula or periscapular soft tissues have traditionally been treated with either a total scapulectomy or a wide, en-bloc, extraarticular scapular resection, termed the "Tikhoff-Linberg" resection. The major challenge following such resections is to restore shoulder girdle stability while preserving a functional hand and elbow.
*Methods:* This report describes three patients who underwent an extraarticular, total scapula resection (modified Tikhoff-Linberg) for a high-grade sarcoma. Each patient was reconstructed with a constrained (rotator cuff substituting) total scapula prosthesis in an effort to optimally restore the normal muscle force couples of both glenohumeral and scapulothoracic mechanisms.
*Results:* At latest follow-up, the MSTS functional score was 24-27/30 (80%–90%). All patients had a stable, painless shoulder and functional hand and elbow. Glenohumeral rotation below shoulder level, shoulder extension and abduction were preserved. Protraction, retraction, elevation and abduction of the scapula were restored and participated in active shoulder motion and upper extremity stabilization. There were no complications.
*Conclusion:* Total scapula reconstruction with a constrained total scapula prosthesis is a safe and reliable method for reconstructing the shoulder girdle following resection of select high-grade sarcomas. This report emphasizes the clinical indications, prosthetic design, surgical technique and early functional results.

**024 Osteosarcoma of the Proximal Humerus: Long Term Results with Limb Sparing Surgery**

James C Wittig[1], Jacob Bickels[2], Kristen L. Kellar-Graney[1], Isaac Meller[2], Martin M Malawer[1]
*[1]Division of Orthopedic Oncology Washington Cancer Institute Washington Hospital Center, [2]National Unit of Orthopedic Oncology Tel-Aviv Sourasky Medical Center*

*Objective:* The purpose of this study was to analyze the long-term oncological and functional results and complications associated with limb-sparing surgery and endoprosthetic reconstruction for 23 patients with osteosarcoma of the proximal humerus.
*Methods:* There were 1 stage IIA lesion, 18 stage IIB lesions, and 4 stage III lesions in this study group. Twenty-two patients were treated with an extraarticular resection that included the deltoid and rotator cuff and one patient was treated with an intraarticular resection that spared the shoulder abductors. In all these patients, the proximal humerus was reconstructed with a cemented endoprosthetic replacement that was stabilized via a technique of static suspension (Dacron tapes) and dynamic suspension (muscle transfers).
*Results:* At latest follow-up (median: 10 years), 15 patients (65%) were alive without evidence of disease. There were no local recurrences. Prosthetic survival was 100% for the 15 survivors. The Musculoskeletal Tumor Society (MSTS) upper extremity functional score ranged from 24 to 27 (80%–90%). All shoulders were stable and pain-free. Elbow and hand function were preserved in all patients. The most common complication was a transient neuropraxia (n=8).
*Conclusion:* En-bloc extraarticular resection and endoprosthetic reconstruction is a safe and reliable method of limb-sparing surgery for high-grade extracompartmental osteosarcoma of the proximal humerus.

**026 Alveolar Soft Part Sarcoma: A Report of 15 Cases**

Serge van Ruth, Frits van Coevorden, Bin B.R. Kroon
*Department of Surgical Oncology Netherlands Cancer Institute, Amsterdam NL*

*Objective:* Alveolar soft part sarcoma is rare tumour representing 0.5–0.9% of all soft tissue sarcomas in adults and 0.8–1.85 of those in children. Published series on presentation and treatment outcome of this sarcoma are scarce. Long-term survival is doubted in most reports. We present our experience with 15 cases, with six, and among them three long-term survivors.
*Methods:* Between 1971 and 2000 15 patients with alveolar soft part sarcoma were headed in our hospital. We collected the files and described their characteristics, therapy and course.
*Results:* The study group consisted of 8 male patients with a mean age at diagnosis of 29 years and 7 female patients with a mean age of 24 years. The primary site was the lower extremity (n=8), buttock (n=2), abdominal wall (n=1), axilla (n=1) and temple (n=1). The median follow-up was 170 (2–370) months. The median survival was 48 months with an overall 5-year survival of 38%. At time of diagnosis 5 patients already had metastases, all pulmonary localized. 5 patients developed early metastases at follow-up, after a tumor free interval of respectively 1, 1, 2, 8 and 12 months. Median survival after diagnosis of metastases was 29 (1–111) months. One is alive with evidence of disease, the other 8 died of their disease. One patient with metastatic disease died after an unexpected long survival (111 months) under various treatment schedules. Another patient with a local recurrence underwent surgery and adjuvant radiotherapy; she is still alive with no evidence of disease, 295 months after the initial diagnosis. Four other patients are alive without disease after 12, 12, 234 and 242 months. In this study chemotherapy influenced the course of the disease only incidentally.
*Conclusion:* Alveolar soft part sarcoma is found especially in young adults. When diagnosed it is often metastasized with a poor prognosis. However, with adequate local treatment, long-term survival is possible.

**029 Surgical Resection, Vascular Reconstruction, and Postoperative Radiation for Leiomyosarcoma of the Inferior Vena Cava**

Fritz Christian Eilber, Gerald Rosen, Michael Selch, Scott Nelson, William Quinones, Frederick Richard Eilber
*Divisions of Surgical Oncology, Medical Oncology, Radiation Oncology, Surgical Pathology and Vascular Surgery, University of California Los Angeles*

*Objective:* Surgical resection for leiomyosarcoma of the inferior vena cava has been minimally effective due to the technical difficulties of surgery, postoperative morbidity, and poor overall survival. The purpose of this study was to determine if aggressive multimodality therapy for leiomyosarcoma of the inferior vena cava would provide survival rates comparable to non-vena caval leiomyosarcomas of the retroperitoneum.
*Methods:* From 1983 to 2000 22 patients with primary leiomyosarcoma of the inferior vena cava were evaluated for multimodality treatment consisting of complete surgical resection, immediate vascular reconstruction of the inferior vena cava and postoperative radiation therapy. Treatment results were reviewed and compared to 41 patients with non-vena caval leiomyosaromas of the retroperitoneum treated at the same institution.
*Results:* Nineteen patients (19/22, 86%) successfully underwent complete surgical resection with vascular reconstruction of the vena cava. The remaining three (3/22, 14%) patients were unresectable due to aorto-mesenteric involvement and died of their disease within 14 months of evaluation. Seventeen of the 19 resectable patients (17/19, 89%) received postoperative radiation and two patients (2/19, 11%) did not. Local tumor control was achieved in 16 of the 17 patients (16/17, 94%) who received postoperative radiation but in neither of the two patients that did not. With a mean follow up of 52 months, the overall survival for the 19 resectable patents was 82% at one year, 53% at three years and 44% at five years. These results are comparable to the 85 % one year, 62% three year and 40% five year survival rate for the 41 patients with non-vena caval leiomyosarcoma of the retroperitoneum treated at the same institution.

*Conclusion:* Complete surgical resection with immediate vascular reconstruction for leiomyosarcoma of the inferior vena cava was achievable in 86% of the cases. This aggressive surgical approach combined with postoperative radiation therapy can provide survival rates similar to non-vena caval leiomyosarcomas of the retroperitoneum and thus provides a successful treatment strategy for a rarely treated malignancy.

**033 Limb-Sparing Endoprosthetic Reconstruction following Major Oncologic Musculoskeletal Resections: Improved Long Term Results and Quality of Life with a Modular System**

Robert M Henshaw, Kristen L. Kellar-Graney, Martin M Malawer
*Division of Orthopedic Oncology Washington Cancer Institute Washington Hospital Center*

*Objective:* Reconstruction of segmental skeletal defects following major resections for sarcoma remains a technically demanding procedure. A modular replacement system (MRS, Pfizer/Howmedica Inc.) was designed as a flexible off-the-shelf solution for resections of the proximal humerus (PH), proximal femur (PF), distal femur (DF), total femur (TF) and proximal tibia (PT). Implant assembly at surgery provides an optimal fit for the bone defect. This study reviews the long-term clinical outcome and prosthetic survival of this system.
*Methods:* 100 consecutive cases since 1986 were retrospectively analyzed. Standard Kaplan-Meier analysis was used to determine prosthetic survival. Failure was defined as any event leading to removal of the implant.
*Results:* Median f/u was 77.8 months (min 2 yrs). Dx at surgery: 72 primary bone sarcomas, 11 metastatic carcinomas, 11 revisions of failed allografts or prior reconstructions, 4 benign aggressive bone tumors, 1 myeloma and 1 lymphoma. Procedures performed: 48 distal femoral replacements, 22 proximal humeral replacements, 15 proximal femoral replacements, 13 proximal tibial replacements and 2 total femoral replacements. Age at surgery: 8 to 78 years. No mechanical failures of the stems, bodies, or interlocking tapers occurred. The ultimate limb-salvage rate was 93% while the infection rate was 8%. Both rates compare very favorably with outcomes associated with other methods of skeletal reconstruction for this patient population. Kaplan-Meier survival analysis of all implants at median f/u of 6.5 yrs was 90.5% [95%CI:0.83%–0.98%]. Survival rates of implants by anatomic sites were: DF 90.3% at median f/u of 76.5 months, PH 95.4% at 91.2 months, PF 100% at 72.2 months, PT 75.1% at 72.2 months, and TF 100% at 38.8 months.
*Conclusion:* Long-term survival and limb salvage rates using the MRS exceeds that reported for allografts, custom endoprostheses, and other modular systems. This system sets a new standard for limb sparing reconstruction following major sarcoma resections as well as allowing limb salvage of failed massive allografts or custom endoprostheses.

**034 Regional Postoperative Analgesia via Indwelling Epineural Catheters Following Major Limb Sparing Resections and Amputations: Analysis of 166 Patients**

Naveh Levy, Robert M Henshaw, Kristen L. Kellar-Graney, Martin M Malawer
*Division of Orthopedic Oncology Washington Cancer Institute Washington Hospital Center*

*Objective:* Continuous postoperative administration of local anesthetics through epineural catheters (surgically implanted within a peripheral nerve sheath, or epineural space) is a novel technique for obtaining regional pain control developed by the senior author over a decade ago. The purpose of this study was to document the safety, efficacy, and reliability of this technique used at a single institution.
*Methods:* Retrospective analysis of 166 pts (median age 45.8 yrs, 86 male/81 female) undergoing limb-saving resection (119), amputation (31), or other major surgical procedure (16) who were implanted with one or more epineural catheters prior to wound closure. Actual nerves implanted with catheters included: sciatic (110), femoral (38), brachial plexus (29), tibial (11), perioneal (7), lumbosacral plexus (4), obdurator (2), median (2), ulnar (1), and tarsal tunnel (1). Following implantation, each catheter was bolused with 10 cc of 0.25% bupivacaine (Marcaine) to achieve an initial block, then continuously infused with bupivacaine for a minimum of 24 hours to a maximum of 21 days post-operatively. 112 pts had complete medication records and were divided into groups: Group 1 pts (34) received an epineural catheter, PCA pump, and a lumbar epidural catheter (triple modality pain control), while Group 2 pts (78) received only an epineural catheter and PCA pump (dual modality pain control). The total amount of narcotics used postoperatively (expressed in mg equivalents of IV morphine), and the verbal mean pain rating (MPR) were recorded.
*Results:* Average MPR and total narcotic use for Group 1 was 2.9 and 320 vs. 3.2 and 240 for Group 2. There were no local complications, i.e. infections, hematomas, or residual neurologic losses, attributable to the implantation or use of epineural catheters. 3 catheters broke at the skin level while being removed; 2 of these catheter tips were removed at bedside while 1 was left *in situ*. 3 catheters were unintentionally pulled out as a result of pt movement. One patient inadvertently died following unintentional infusion of a marcaine dosage though an IV line instead of the catheter.
*Conclusion:* (1) The use of epineural catheters following limb sparing or ablative surgery provides dramatic postoperative analgesia comparable to that seen in pts with epidurals. (2) This technique is simpler, safer and more reliable than epidural catheters without any risk for epidural hematoma, abcess, or spinal leak, particularly in pts who have recently received chemotherapy. (3) We recommend epineural catheters as a primary mode of postoperative pain control following major resections and amputations at the pelvic, shoulder girdle level as well at the hip and knee joint.

**039 The Concept of a Gluteal (Buttock) Compartment: Radiopathological Considerations, Indications, and Surgical Technique of Resection**

James C Wittig[1], Felasfa Wodajo[1], Jacob Bickels[2], Yehuda Kollender[2], Kristen L Kellar-Graney[1], Robert M Henshaw[2], Isaac Meller[2], Martin M Malawer[1]
*[1]Division of Orthopedic Oncology Washington Cancer Institute Washington Hospital Center, [2]National Unit of Orthopedic Oncology Tel-Aviv Sourasky Medical Center*

*Objective:* This study reports our guidelines for limb-sparing surgical resection of gluteus maximus tumors in lieu of hemipelvectomy and presents our oncological functional results. The concept of a buttock compartment is presented with its unique pathologic and radiologic considerations.
*Methods:* Between 1991 and 2001, 13 patients were treated (7 males, 6 females) with buttock resection. Histology included: liposarcoma (5), leiomyosarcoma (3), MFH (1), fibromatosis (2), and myxoma (2). Median tumor size was 10 cm. Six patients received adjuvant chemotherapy or radiation. Surgical resection was performed through a curvilinear incision in the shape of a question mark. The sciatic nerve was explored early and frozen section biopsy performed through its fascial sheath. The inferior gluteal vessels and branches of the superior gluteal vessels were ligated at the level of the sciatic notch and the gluteus maximus was resected *en bloc*.

*Results:* The follow-up ranged from 2 months to 80 months (mean: 30.5 months). There were no local recurrences. Three patients died of metastatic disease. Pathological analysis demonstrated the sciatic nerve sheath to be free of tumor and the deep fascia of the gluteus maximus to be intact in all patients. All patients were ambulatory without assistance. No patient reported compromise in functional activities. Two patients complained of pain when sitting on hard surfaces for prolonged periods of time. Complications included Skin Flap Necrosis (partial) in 4 patients.
*Conclusion:* Surgical resection of the buttock is a safe and reliable surgical option for treatment of selected soft-tissue tumors arising from the gluteus maximus. Gluteus maximus tumors can grow to large sizes but remain contained within the investing fascia of the muscle, thus facilitating compartmental resection by single myectomy. There is minimal functional loss following resection of the gluteus maximus.

**048 The Influence of Anatomical Location on Outcome in Extremity Soft Tissue Sarcoma**

C H Gerrand[4], R S Bell[2], J S Wunder[1], R A Kandel[1], B O'Sullivan[2], C N Catton[2], A M Griffin[1], A M Davis[3]
*[1]University Musculoskeletal Oncology Unit, Mount Sinai Hospital and the University of Toronto, [2]Princess Margaret Hospital of the University Health Network, [3]Toronto Rehabilitation Institute and University of Toronto, [4]North of England Bone and Soft Tissue Tumour Service, Freeman Hospital*

*Objective:* To determine if the rates of local recurrence and metastasis differ in upper versus lower extremity sarcomas.
*Methods:* Prospectively collected data relating to patients who underwent limb-sparing surgery for extremity soft tissue sarcoma between January 1986 and April 1997 were analysed. The local recurrence-free and metastasis-free rates were calculated using the method of Kaplan and Meier. Univariate and multivariate analyses of potential predictive factors were evaluated with the log-rank test and the Cox proportional hazards model.
*Results:* Of 480 eligible patients, 48 (10.0%) had a local recurrence and 131 (27.3%) developed metastases. The median follow-up of survivors was 4.8 years (0.1 to 12.9). There were 139 upper and 341 lower extremity tumors. Upper extremity tumors were more often treated by unplanned excision before referral than lower extremity sarcomas (89 (64.0%) vs. 160 (46.9%), p=0.001) and were smaller (6.0cm vs 9.3cm, p=0.000). Lower extremity tumors were more often deep to or involving the investing fascia (280 (82.9%) vs. 97 (69.8%), p=0.003). The distribution of histological types differed in each extremity. Fewer upper extremity tumors were treated with adjuvant radiotherapy (98 (70.5%) vs. 289 (84.8%), p=0.000). The local recurrence-free rate at five years was 82% in the upper and 93% in the lower extremity (p=0.002). Local recurrence in the Cox model was predicted by surgical margin status (hazard ratio 3.16, p=0.000) but not extremity (p=0.127) or unplanned excision before referral (p=0.868). The metastasis-free rate at five years was 82% in the upper and 69% in the lower extremity (p=0.013). Metastasis was predicted by high histological grade (hazard ratio 17.28, p=0.000), tumor size in cm (hazard ratio 1.05, p=0.001) and deep location (hazard ratio 1.93, p=0.028) but not by extremity (p=0.211).
*Conclusion:* Local recurrence is more frequent after treatment for upper compared with lower extremity sarcomas. Variation in the use of radiotherapy and differences in histological type may be contributory. Metastasis is more frequent after treatment for lower extremity sarcomas because tumors tend to be large and deep compared with upper extremity tumors.

**053 Curettage Resection and Cryosurgery for Extremity Low Grade Chondrosarcoma**

Felasfa Wodajo, Kari L Mansour, James C Wittig, Frank H Kim, Robert M Henshaw, Martin M Malawer
*Department of Orthopedic Oncology Washington Cancer Institute Washington Hospital Center*

*Objective:* Previously reported treatments for low-grade chondroid tumors have ranged from simple curettage to wide resection to even amputation. Curettage resection for cartilage tumors, while attractive for being associated with less morbidity than resection has been criticized as leading to higher recurrence rates. For the past 13 years, we have used cryosurgery as an adjunct to curettage to eradicate residual microscopic disease in an effort to improve local tumor control over curettage alone. The purpose of this study is to report the oncological and functional outcome of extremity low grade chondrosarcoma treated with consistent surgical indications and techniques.
*Methods:* Curettage resection for cartilage tumors, while attractive for being associated with less morbidity than resection has been criticized as leading to higher recurrence rates. We have used cryosurgery as an adjunct to curettage to eradicate residual microscopic disease in an effort to improve local tumor control over curettage alone. The purpose of this study is to report the oncological and functional outcome of extremity low grade chondrosarcoma treated with curettage, cryosurgery and cemented internal fixation.
*Results:* Average length of follow-up was 73 months (range 25–161 months). There were no local recurrences or distant metastases. Functional results in the proximal humerus were as follows: 14/23 good (i.e. minimal pain and/or minimal limitation of motion), 6/23 excellent (no pain, normal range of motion) and 3/23 fair. In the distal femur, outcomes were 2/16 excellent, 13/16 good and 1/16 fair. Complications included one 70 year-old patient who had late (3 year) post-operative degenerative changes of the shoulder underwent shoulder arthroplasty. One non-displaced unicortical fracture in the distal femur was treated successfully without immobilization. There was one case of reflex sympathetic dystrophy of the upper extremity which resolved with sympathetic blockage.
*Conclusion:* Curettage resection and cryosurgery for low grade chondrosarcoma, if applied according to strict patient selection criteria and followed by appropriate reconstructive techniques allows for preservation of near-normal limb function with minimal rates of complications and recurrence. We do not recommend *en bloc* resection for the treatment of low grade extremity chondrosarcoma.

**058 Excisional Biopsy Prior to Wide Resection of Extremity Sarcoma is Associated with Improved Outcome**

Mark D. McKee[1], Brian P. Whooley[2], Deborah Driscoll[1], John F. Gibbs[1], William G. Kraybill[1]
*[1]Roswell Park Cancer Institute, [2]Saint Vincent's Hospital and Medical Center*

*Objective:* Needle or incisional biopsies are preferred over excisional biopsy for suspected sarcoma since they are limited procedures, they minimize the amount of surrounding tissue exposed to tumor, and the sites are easily encompassed at the time of wide resection. Sarcoma treatment by definitive wide resection after excisional biopsy (re-resection) has been reported to independently predict freedom from distant metastases and improved overall survival. We evaluated patients undergoing surgery for sarcoma to determine the influence of excisional biopsy and re-resection on patient outcome.
*Methods:* Clinical factors, pathologic factors, and outcome were determined for 202 patients who were surgically treated for extremity soft tissue sarcoma between 1982 and 1998. Outcomes were measured for patients undergoing definitive surgery after excisional biopsy (re-resection) or definitive surgery after incisional, needle, or no biopsy (single resection).
*Results:* Tumors were predominantly high-grade (148/196), larger or equal to 5cm in size (142/200), deep (166/202), and located on the lower limb (145/202). Treatment included wide local excision, compartment resection, or amputation. Final resection margins were negative for 190/202. Patient / tumor factors: Patients who underwent re-resection were more likely to have small tumors, superficial tumors, upper extremity tumors, and be of younger age. Gender, duration of symptoms, tumor grade, histology, and resection margin were no different between groups. Outcome: Recurrence and survival by group are shown in the table below. Margin status after excisional biopsy did not influence patient outcome. Multivariate analysis: Factors associated with improved overall survival (OS) by univariate analysis included young patient age (p<.001), low tumor grade (p<.001), small tumor size (p<.01), superficial location (p<.05), and re-resection (p<.001). By multivariate analysis, improved disease-free survival (DFS) was predicted by low tumor grade (p<.01) and re-resection (p<.05). Improved OS was predicted by low tumor grade (p<.01) and re-resection (p<.001).

| | N | LR (p=NS) | DR (p<.001) | 5 yr DFS (p<.001) | 5 yr OS (p<.001) |
|---|---|---|---|---|---|
| Re-resection | 109 | 17 (16%) | 26 (24%) | 60% | 72% |
| Single resection | 93 | 18 (19%) | 43 (46%) | 26% | 48% |

*Conclusion:* Re-resection following excisional biopsy for extremity sarcoma independently predicted freedom from distant recurrence and improved survival. These results were unexpected, and could not be entirely explained by differences in clinico-pathologic features between the groups. The biologic reasons for improved outcome among patients undergoing re-resection are not clear.

### 065 The Value of Population Based Research in Soft Tissue Sarcoma (STS)

Chris J de Gara, Juanita Hatcher, Doug Dover, George Dundas, Guy Lavoie, Robert Turner
*The Cross Cancer Instiute, University of Alberta*

*Objective:* Because of the heterogeneity and rarity of STS, Level I evidence is largely lacking and this results in a variety of treatment approaches. Population Based Outcomes Research may be of value in STS management as it is generalizable, includes and follows all patients and avoids the selection, referrer and other biases inherent in case series and even randomized study.
*Methods:* The Alberta Cancer Registry, by law, registers all cancers including STS, patient demographics, tumour and treatment details and overall survival. There are two Regional Cancer Centres (Tom Baker in Southern Alberta population 1.3M and the Cross Cancer Institute in Northern Alberta population 1.7M).
*Results:* Between 1990 and 1999, 380 patients (53% male; 211 Northern and 169 Southern) over the age of 18 years (mean 56 + 18 years) were diagnosed with STS. The most common tumours were MFH (30%), sarcoma NOS (27%), leiomyosarcoma (16%) and liposarcoma (15%) with no significant differences between centres. Other than pelvis n = 33 (Northern) vs. 16 (Southern) p = 0.01 and trunk n = 17 (Northern) vs. 6 (Southern) p = 0.02, tumour location was similar in Northern and Southern Alberta. Northern Alberta coded significantly more high grade tumours (41%) than Southern Alberta (18%) p 0.01. Significant treatment differences existed between Northern and Southern Alberta: surgery + radiation + chemotherapy 5% vs. 38% p 0.0001, surgery + radiation 30% vs. 11% p 0.0001, and surgery alone 46% vs. 29% p 0.0001. These differences did not result in survival differences. For limb, 5 year survival was 69% (Northern) vs. 66% (Southern) and for non-limb was 56% (Northern) vs. 52% (Southern). These differences were not significant. For the province as a whole the overall 5 year non-limb survival was 54% vs. limb 67% p = 0.0072.
*Conclusion:* In summary, using a population based approach to the assessment of STS within a province, differences in treatment and tumour grade were not associated with overall survival differences. While local recurrence and quality of life data are not yet available, we concluded that this approach can provide valuable insights into the management of this complex cancer.

### 068 Atypical Lipomas of the Extremities

Tamara Rosenthal, Lisa Khoury, Rakesh Donthineni-Rao, Richard Lackman
*Department of Orthopaedics, University of Pennsylvania*

*Objective:* Atypical lipomatous tumors also known as well differentiated liposarcomas occur predominantly in middle-aged patients and often present to the orthopaedic surgeon as painless, slow-to-moderately fast growing masses in the extremities. The purpose of this study is to describe the natural history and treatment outcome of 31 patients with superficial or deep atypical lipomas of the extremities.
*Methods:* The records of patients treated for atypical lipomas of the extremities were reviewed. Criteria for inclusion were a diagnosis of superficial or deep atypical lipomas made by surgical pathology, as well as a minimum follow-up of one year from the time of original diagnosis.
*Results:* Thirty-one patients met the inclusion criteria for this study. There were 16 males and 15 females, with an average age of 57 years at the time of the initial presentation (range 32–87 years). Mean follow-up was 7 years (range 1–28.8 years). At presentation, 19 patients reported a slowly growing mass and 12 patients reported pain as the initial symptom. Twenty-five tumors occurred in the lower extremity and 6 in the upper extremity. Sixteen patients (52%) had a recurrence at an average of 4.7 years after resection (range 2 months–10 years). Twelve (39%) patients required additional surgical procedures to treat their tumor. Dedifferentiation to high-grade liposarcoma developed in three patients (10%). These were treated with resection and radiation with or without chemotherapy, and are disease free at latest follow-up.
*Conclusion:* Atypical lipomas, whether deep or superficial, have a high propensity for local recurrence and a potential for malignant dedifferentiation. These tumors thus require careful evaluation, adequate surgical treatment and close clinical follow-up extending beyond five years.

### 069 Epithelioid Sarcoma: Clinical Behaviour of a Rare Soft Tissue Sarcoma

Dario Baratti, Alessandro Gronchi, Paolo Casali, Laura Lozza, Elisabetta Pennacchioli, Rossella Bertulli, Palma Dileo, Mario Santinami
*Istituto Nazionale per la Cura e lo Studio dei Tumori*

*Objective:* Epithelioid sarcoma (ES) is a rare subtype of soft tissue sarcoma (STS). Distinctive clinical features have a tendency to be multifocal at presentation and to spread locally at recurrence, with an higher rate of lymph nodes involvement and in-transit metastasis.
*Methods:* Twenty-nine consecutive patients with ES were observed at our institution from June 1985 to June 2001. Twenty-two were male and 7 female; median age at the time of first diagnosis was 29 years (range 9–66). Site of first presentation was the hand in 12 cases, the upper limb in 5, the foot in 2, the lower limb in 6 and the trunk in 4. Fourteen out of 29 patients referred after a median of 3 consecutive relapses (range 1–5), developing multifocal recurrences in 4 cases, loco-regional nodal involvement in 2

and distant metastasis in 1. Two patients underwent amputative surgery before our observation. Primary treatment of choice performed in our department was wide en-bloc excision, with the aim to obtain adequate margin of uninvolved tissue in all directions, and to spare limbs and limb function as much as possible. Adjuvant RT was administered to high risk patients in a postoperative fashion.

*Results:* ES presented as small superficial nodules in 18 cases and this feature was prevalent in distal localization (11/14), while deep-seated larger masses were more common in the proximal extremities or in the trunk (8/15). Surgical treatments performed were: wide excision in 15 patients, re-excision in 8, digit or ray amputation in 3, major amputation in 3. Surgical margins were rated as wide in 16 patients and marginal in 5. No residual tumor was found in any patient who underwent re-excision. Adjuvant therapies were the following: postoperative RT in 8 patients, preoperative limb perfusion in 5, preoperative conventional CT in 4 and postoperative CT in 1. Median follow-up of the series was 32 months (range 3–181). Actuarial overall survival at five years was 76.5%; disease free survival at the same interval was 55.8%. Following treatment at our Institution, from 1 to 4 consecutive relapses (median 2) occurred in 12 patients. Recurrence involved loco-regional lymph nodes in five more patients, thus overall lymph nodal involvement rate was 7 out of 29 (24.1%). Distant metastases were detected at the time of first presentation in one case, developed as first recurrence in two and was subsequent to nodal involvement in six. Most common site of distant metastatization was the lung. All the patients with metastatic disease have died. Four more amputations were needed to treat local recurrence, thus a total number of 12 such procedures were performed in 8 patients. Relapse occurred in 3 out of 15 (20%) patients treated by primitive neoplasm and in 9 out of 14 (64.3%) treated for recurrent tumor.

*Conclusion:* Epithelioid sarcoma is a rare neoplasm, accounting for less than 1% (29/3376) of all the STS observed during the period of the study. The present study showed a great propensity to recur repeatedly and an high rate of nodal involvement was confirmed. Lack of local control lead to an increase in amputation rate. Adequacy of treatment was the most relevant prognostic factor, since patients referred for recurrent disease, after repeated attempts of local control, had a worse outcome than patients that underwent primary treatment in our department.

**079 Two Hundred and Seven Extra-Abdominal Aggressive Fibromatosis Treated at a Single Institution: An Analysis of Outcome.**

Alessandro Gronchi[1], Dario Baratti[1], Elisabetta Pennacchioli[1], Luigi Mariani[2], Salvatore Lo Vullo[2], Laura Lozza[3], Maurizio Colecchia[4], Paolo Casali[5], Rossella Bertulli[5], Mario Santinami[1]
[1]*Dept. of Muscolo-skeletal surgery. Istituto Nazionale per lo studio e la cura dei Tumori.*, [2]*Dept. of Biostatistics. Istituto Nazionale per lo studio e la cura dei Tumori.*, [3]*Dept. of Radiotherapy. Istituto Nazionale per lo studio e la cura dei Tumori.*, [4]*Dept. of Pathology. Istituto Nazionale per lo studio e la cura dei Tumori.*, [5]*Dept. of Medical Oncology. Istituto Nazionale per lo studio e la cura dei Tumori.*

*Objective:* Aggressive Fibromatosis (AF) is a rare benign disease, characterized by a high local failure rate, with possible implication in quality of life and sometimes even risk of death. A series of 207 patients, treated at our institution over a 30 year span and followed prospectively, has been reviewed to find out possible predictive criteria of failure.

*Methods:* From May 1966 to February 2001 207 pts (median age 36, M/F 51/156), affected by AF (arising from extremities in 41, girdles in 66, abdominal or chest wall in 56 and from other sites in 44), have been treated at our institution. 131 (median size 5 cm) presented with primary disease, while 76 (median size 6 cm) had had at least one local recurrence before being referred. Surgery alone in 167 patients, while surgery + radiation therapy in 40 (20 for each group) has been the procedure performed at presentation. Quality of margins was reviewed by a single pathologist and 149 were found to be negative (99 for primary cases and 50 for recurrences), while 58 were microscopically positive (32 and 26 respectively). Both univariate and multivariate (Cox model) analyses of possible prognostic factors were performed.

*Results:* During follow-up (median time 85 months) 55 patients recurred. Four patients died of local progression. The overall survival was 98% at 5 years and 95% at 10 years, while disease free survival was 71% at 5 years and 68% at 10 years. Primary cases had better outcome with 80% disease free survival at 5 years and 74% at 10 years. Recurrences had 56% disease free survival at 5 years, stable up to 10 years. In primary cases size (> 5cm) and site (extremities and girdles) were found to be significant prognostic factors, while quality of margins did not affect outcome. In recurrences none of the prognostic factors analyzed were found to be significant in predicting local recurrence. Multivariate analysis confirmed the prognostic role of status at presentation (primary versus recurrence) and size. A borderline P was obtained for site and there was a suggestion that quality of margins could be prognostic only in recurrences.

*Conclusion:* At variance with sarcomas, quality of margins doesn't predict outcome in primary tumors. Wide resection is still the cornerstone of treatment, but attempts to achieve negative resection margins may result in unnecessary morbidity and may not prevent local recurrence. Operations that preserve function and structure should be the primary goal, because the presence of residual disease cannot be clearly shown to impact adversely on 5 years and 10 years disease free or overall survival. Recurrences are at a higher risk of further recurrence, especially if operated with positive resection margins. Adjuvant radiation may be delivered in selected cases.

**077 Long Term Follow-Up of Stage IIB Extremity Osteosarcoma: The Effect of Local Tumor Extent on Prognosis**

Jose M Casanova[2], Susanne S Spanier[1], Robert E Leggon[3], Mark T Scarborough[1]
[1]*University of Florida*, [2]*Hospitais da Universidade de Coimbra*, [3]*Geisinger Medical Center*

*Objective:* More recent treatment of osteogenic sarcoma (OGS) has improved early survival, yet few protocols utilizing surgery and chemotherapy (chemo) have follow-ups (fu) approaching 20 yrs. We wished to test the hypotheses that: 1) relapses and treatment-related deaths occurred 10 years after initial treatment, decreasing survival; 2) the amount of initial local tumor extension remained a prognostic factor; and 3) the initial size of the tumor remained a prognostic factor; all with a fu approaching 20 yrs.

*Methods:* In 1999 we re-examined the data of 71 pts with high grade OGS who were treated at the University of Florida from Jan 1, 1979 to Aug 1, 1984. All 71 had surgery, 69 had chemo, and 64 had radiation (59 whole lung, 5 local). 62 of 71 had extremity lesions. Evaluation included 59 pts with conventional, Stage IIB OGS of the extremities: 51 followed Protocol (Prot) (surgery, 5 courses of postoperative Adriamycin (total dose of 450 mg/m2), and 1600 centigray of whole-lung irradiation); 8 did not. Limb salvage surgery was performed in 31% and amputation in 69% of the Prot pts. Tumor extension was graded E1 to E6 for protocol pts, and the maximum tumor dimension was recorded. Tumor extension was E1-E5 (only touching periosteum-invading one structure) in 26 pts and E6 (invading two or more structures) in 25 pts [ref 1]. The probability of overall survival, according to the Kaplan-Meier (K-M) method, was plotted for: all pts (n=71); all chemo pts (n=69); extremity pts (n=62); all extremity IIB pts (n=59);

Prot pts (n=51); Prot E1-E5 pts (n=26); Prot E6 pts (n=25); non-extremity pts (n=9); and Prot violators (n=8). K-M curves were compared using a log-rank test, and survival relative to tumor size was evaluated with a t-test. [ref 1. Spanier, S.S.; Shuster, J.J.; and Vander Griend, R.A.: The effect of local extent of the tumor on prognosis in osteosarcoma. *J Bone Joint Surg.*, 72-A:643-653, 1990.]
*Results:* Average fu was 17.4 yrs for survivors. Survival at 5, 10, 15, and 20 years was: 66,54,54,52% for all pts (n=71); 67,55,55,53% for all chemo pts (n=69); 64,57,57,57% for all extremity pts (n=62); 64,57,57,57% for all extremity IIB pts (n=59); 61,55,55,55% for all Prot pts; 81,73,73,73% for all Prot E1-E5 pts (n=26); 40,36,36,36% for all Prot E6 pts (n=25); and 78,33,33,22 for non-extremity pts (n=9). A statistically significant difference in survival remained between groups Protocol E1-E5 and Protocol E6 (p=0.005). Tumor size (av) was 9.4 cm for survivors; 11.6 cm for those dead of disease (p=0.06).
*Conclusion:* 1) Few late relapses and treatment related deaths occurred greater than 10 yrs after initial treatment; 2) the initial local tumor extension (E1-E5 vs E6) remained a statistically significant prognostic indicator; while 3) tumor size was only marginally significant with a fu approaching 20 yrs.

### 085 Close Surgical Margins in Soft Tissue Sarcoma Resection Do Not Predict Local Recurrence When Induction (Neoadjuvant) Chemotherapy is used in the Treatment of High Grade Extremity Soft Tissue Sarcoma

Felasfa M. Wodajo, James Wittig, Kari Mansour, Dennis Priebat, Robert Henshaw, Martin Malawer
*Washington Cancer Institute Washington Hospital Center*

*Objective:* Surgical resection with "wide" margins, i.e. with a rim of normal of tissue, is the mainstay of local control for high grade soft tissue sarcoma. The objective of this study is to assess whether the presence of close or microscopically positive surgical margins contributes to local recurrence and what role, if any, induction chemotherapy without preoperative radiation plays in prevention of local recurrence after limb-sparing resection of soft tissue sarcomas.
*Methods:* During the period of 1988 to the present, over 100 resections for high-grade soft tissue sarcomas were performed by the same surgical team. Of these, 58 whose tumors were deep, large (more than 5 cm) and high grade were eligible for enrollment into a chemotherapy protocol consisting of neoadjuvant plus adjuvant doxorubicin, intra-arterial cisplatin and, after 1996, ifosfamide. Patients did not undergo preoperative radiation but those with poor chemotherapeutic response and close surgical margins also underwent adjuvant external beam radiotherapy. Twenty nine patients who underwent their first surgery after induction chemotherapy and have follow-up of two years or more form the basis of this report. Pathology reports from 15 of these cases specify a margin depth in centimeters.
*Results:* The overall local recurrence rate was 6.9% (2/29). For the 27 patients without local recurrence, 14 (52%) have pathology reports specifying margin depth. Of these 10/14 (71%) had a minimum distance of less than or equal to 5 mm from the tumor to the inked margin whereas 1/14 (7%) had a "positive" margin, where tumor is focally present at the inked margin. Among the 2 patients with local recurrence, 1 (50%) has a pathology report specifying the margin depth which was less than or equal to 5 mm. Pathology reports not specifying margin depth stated generally that the margins were "free of tumor". Adjuvant radiotherapy was administered to 1 (50%) patient with local recurrence (margin depth not specified) and 3 (11%) patients without local recurrence all of whom had 20% or less tumor necrosis (one margin less than 5 mm, one margin unspecified). The maximum tumor diameter in non-recurring and recurring patients averaged 11.2 cm and 15.8 cm, respectively.
*Conclusion:* Even with the use of adjuvant radiotherapy, several recent large reviews have reported 20%–25% local recurrence rates with the most important risk factor being the surgical margins of the resection specimen. In our series, there was only a 7% local recurrence rate which is even more remarkable when it is noted that, of the patients without local recurrence who had exact margins recorded, nearly 80% had a surgical margin of 5mm or less. Thus the presence of microscopically close or positive resection margins in our series did not correlate with local recurrence. Furthermore, only 4/29 (14%) patients underwent postoperative radiotherapy and no patient underwent preoperative radiotherapy suggesting that radiation did not significantly contribute to this low recurrence rate. While systemic chemotherapy is generally administered in an attempt to improve overall survival, our results imply that the killing of malignant cells in the periphery of tumors with induction chemotherapy can improve local control and postoperative function by allowing for the removal of a minimum amount of normal tissue with an acceptable rate of local recurrence.

### 088 Spermatic Cord Sarcoma: Outcome, Patterns of Failure and Management

Matthew T. Ballo, Gunar K. Zagars, Peter W. T. Pisters, Barry W. Feig, Shreyaskumar R. Patel, Andrew C. von Eschenbach
*UT M.D. Anderson Cancer Center*

*Objective:* To evaluate the outcome, elucidate the patterns of failure and suggest treatment strategies for sarcoma arising in the spermatic cord.
*Methods:* Between 1956 and 1998, 32 patients were identified through a search of our patient registry with the diagnosis of non-metastatic spermatic cord sarcoma. A retrospective review of disease outcome, patterns of relapse and patient survival was performed.
*Results:* Histologic subtypes of sarcoma were: malignant fibrous histiocytoma (12 patients), leiomyosarcoma (6 patients), liposarcoma (8 patients), and other subtypes (6 patients). All patients had radical orchidectomy with or without additional resection to achieve negative margins. Margins were microscopically negative in 29 and positive in three. Three patients received adjuvant radiation to the surgical site. With a median follow-up of 9 years, 14 patients have died resulting in a 10- and 15-year actuarial overall survival of 63% and 52%, respectively. The actuarial 10- and 15-year local control, distant metastasis-free and disease-free survival rates were 72% and 61%, 85% and 85%, and 60% and 51%, respectively. Pattern of failure analysis revealed 8 local failures, 2 regional failures within the pelvis and 6 distant failures. Of the 6 distant failures 2 included synchronous para-aortic lymph node metastases resulting in an actuarial 15-year para-aortic failure rate of 7%. Only three of the seven patients in whom disease recurred locally were salvaged. None of the three patients treated with combined surgery and radiation relapsed.
*Conclusion:* Spermatic cord sarcoma has a high propensity for local recurrence following surgery, while para-aortic nodal failures remain uncommon. This result suggests a role for localized radiotherapy to the pelvis. Elective surgery or irradiation of the para-aortic lymph nodes appears unnecessary.

### 094 Neoadjuvent Adriamycin and Twice Daily Radiotherapy in Adult Soft Tissue Sarcoma

John Heiner, Minesh Mehta, Howard Bailey, Jack Fowler, Timothy Kinsella, Michael Lamson, F. Kristian Storm
*University of Wisconsin Medical School Department of Surgery – Surgical Oncology, Orthopedics, Biostatistics and the Department of Human Oncology – Radiation Therapy, Medical Oncology*

*Objective:* A series of limb salvage trials at UCLA employing preoperative continuous infusion of adriamycin (ADR), either intraarterially (IA) or intravenously (IV), followed by daily radiotherapy treatments were performed. External beam radiation (XRT) to total doses of 35 Gy (3.5 Gy x 10), 28 Gy (3.5 Gy x 8), or 17.5 Gy

(3.5 Gy x 5), were analyzed with the findings that both local recurrence and wound complications were radiation dose dependent but independent of the route of ADR administration. ADR and 28-35 Gy XRT daily over 8–10 days resulted in effective limb salvage (96%–100%) with 5%–10% local recurrence (LR) at 36 month median, but there were 10%–23% major wound complications requiring reoperation. We postulated that twice-daily doses of lower fraction size XRT might achieve a similarly low incidence of local recurrence with a further reduction in postoperative wound complications.

*Methods:* The patients in Group 1 (n=10) were treated with intraarterial adriamycin by continuous infusion to a total dose of 90 mg over a 3 day period. The patients in Group 2 (n=43) were treated with the same dose intravenously. Radiation was begun 1 to 2 days after the completion of chemotherapy. The patients in Group 1 were treated with 3.5 Gy qd X 8 days. The patients in Group 2 were treated with 2 Gy BID X 8 days. Patients were evaluated for perioperative wound complications using the late effects on normal tissues scoring system. Patients were also evaluated for local recurrence.

*Results:* 53 patients with Grade IIB and Grade IIIB lesions underwent treatment. Average follow-up was 47 months. Average greatest tumor diameter was 8.00 cm in Group 1 and 8.82 cm in Group 2. There was 1 (10%) local recurrence in Group 1 and 1 (2.33%) local recurrence in Group 2. There were 3 major complications (30%) and 2 minor complications (20%) in Group 1. There were 2 (4.65%) major complications and 9 (20.93%) minor complications in Group 2. This suggested a decreased complication rate with twice daily dosing however statistical significance was not achieved (p=0.091).

*Conclusion:* We interpret these results to indicate that preoperative IV-ADR and 28-32 Gy XRT over 8 days results in effective limb salvage and similarly low rates of LR, but that 2.0 Gy delivered twice daily to a total dose of 32 Gy appears to lower the incidence of postoperative complications.

**095 What should be the Surgical Treatment of Gists? Research on 18 Patients with Gists**

Takeo Sato[1], Matthias Peiper[1], Thomas Storeichert[1], Annette Fritscher-ravens[2], Wolfram-Trundo Knoefel[1], Nib Soehendra[2], Jacob R. Izbicki[1]

*[1]Dept. of General Surgery, University of Hamburg, [2]Dept. of Endoscopy, University of Hamburg*

*Objective:* The diagnosis and treatment of gastrointestinal stromal tumors (GISTs) is still a field of intensive investigation. Since 1990 we performed exact clinical examination of GISTs of the stomach which were treated over ten years in this hospital, we report.

*Methods:* This is a retrospective study of 18 patients with GIST that were treated in our hospital from 1990 to 1999. The documented parameters were diagnosis especially endosonography, surgical treatment, pathological diagnosis, TNM classification, and the prognosis.

*Results:* 9 males and 9 females with a mean age of 61 years (34–83). In most of the cases the symptoms were unspecific. 13 patients suffered from nausea, 3 patients had stomach pain, and GI-bleeding was present in 2 patients. The diagnosis was achieved by endoscopy and endosonography, a biopsy was taken in 2 cases. One of them showed a PAPll and other a PAPlll lesion. Liver metastasis was found in 2 patients before the operation. 8 patients received a total gastrectomy with splenectomy, 5 patients a distal gastrectomy, a gastric fundectomy was performed in 2 patients and 3 patients were treated by local resection. The 2 patients showing liver metastasis received a liver excision. The postoperative pathological diagnosis revealed in all of these cases GISTs. The lymph node metastasis was found to one patient. The average of tumor size was 10.3cm. 5 patients were T1, and 13 patients showed T2. 10 patients were G1, 4 patients showed G2, and 4 patients were G3. R0 was performed in 17 patients, and 1 patient was received R1. In 2 cases metastasis was found after operation, one of them showed peritonitic carcinomatosis. The death due to GIST was shown with four cases and the mean survival term was 52 months (range 4–98).

*Conclusion:* Our data suggests that in absence of criteria for malignancy the resection of submucosal tumors is not indicated. GISTs cause metastasis only in few cases while metastasis to distant lymph nodes is very rare. We perform close controls in these patients, in suspicious cases or if complications occur we performed a resection.

**097 Isolated Limb Infusion: A Novel Treatment for Locally Advanced Soft Tissue Sarcoma of the Extremity**

Robert C.G. Martin, Karen Brown, Mary S. Brady

*Memorial Sloan-Kettering Cancer Center Department of Surgery*

*Objective:* Isolated limb infusion (ILI) is a technique of delivering regional chemotherapy to the extremity in patients with advanced melanoma or soft tissue sarcoma (STS). ILI is performed by administering high doses of chemotherapy into a normothermic, hypoxic, and low flow circuit via angiographically placed catheters after a tourniquet is placed around the proximal extremity. Unlike the standard approach of isolated limb perfusion (ILP), no surgical incision is required. In addition, the procedure is less time consuming, lends itself to repeat treatments and is associated, in our initial experience, with a shortened hospital stay and less morbidity than conventional ILP.

*Methods:* We report our experience with a 60 year old man who presented with a rapidly growing, high grade STS of the calf. The tumor was unresectable other than by amputation due to encasement of the nerves and vasculature. The patient was treated with neoadjuvant chemotherapy (high dose adriamycin and ifosfamide) and progressed on this regime (see figure). The patient underwent ILI with melphalan and dactinomycin and had significant tumor shrinkage resulting in restoration of his dorsalis pedis pulse. A second ILI was performed 10 weeks later resulting in a decrease in the dimensions of his tumor by MRI from 12.2cm x 7.8cm prior to the first infusion to 5.5cm x 7.3cm 8 weeks after his second (see figure). The patient remains without systemic disease, and 23 weeks after his initial ILI has experienced no significant morbidity from his regional therapy.

*Results:* Figure: Tumor volume by MRI in relation to treatment.

*Conclusion:* ILI using melphalan and dactinomycin may be an effective means of regional control in patients with unresectable STS with less morbidity than ILP.

**107 Predictive Factors for Wound Complications following Free and Pedicled Soft Tissue Flaps for Limb Reconstruction After Excision of Soft Tissue Tumours**

A T Abudu[1], R S Bell[1], A M Griffin[1], B O O'Sullivan[2], C N Catton[2], A M Davis[1], J S Wunder[1]

*[1]University Musculoskeletal Oncology Unit, Mount Sinai Hospital, University of Toronto, [2]Department of Radiation Oncology, Princess Margaret Hospital*

*Objective:* The literature on the outcome of patients with soft tissue sarcoma who require soft tissue reconstruction with free or pedicled flaps is scanty. We investigated the risk factors for wound complications in patients with soft tissue sarcoma of the extremity who required reconstructive soft tissue flaps. We also compared the local oncologic control in patients requiring reconstructive flaps with those in whom primary wound closure was possible.
*Methods:* Prospectively collected database records and the clinical data of all the patients who underwent excision of soft tissue sarcoma of the extremity and reconstruction with a free or pedicled soft tissue flaps at our centre were retrospectively reviewed. Wound complication was defined as a complication at the site of tumour excision necessitating a return trip to the operating room or prolonged wound packing. Chi-square test was used for analysis of nominal data.
*Results:* 113 consecutive patients with soft tissue sarcoma who had limb preserving surgery with soft tissue reconstructive flaps were studied. The minimum follow-up was 24 months. The mean age was 55 years (16–95). The sarcoma was located in the lower extremity in 83 and upper extremity 30 patients. Adjuvant radiotherapy was administered pre-operatively in 64 patients, post-operatively in 31, brachytherapy in 4 and no radiotherapy in 14 patients. Significant wound complications developed in 37 patients (33%). The most common complications were wound infections or partial necrosis occurring in 16% (18/113) and 13% (15/113) respectively. Complete flap necrosis requiring flap removal occurred in 6 patients (5%). Three patients (2.3%) required amputation as a result of the complications. The statistically significant risk factors for development of wound complications include location of tumour in the lower limb compared to upper limb (relative risk 2.3, p=0.02) and use of pre-operative radiotherapy compared to no or post-operative radiotherapy (relative risk 2.05, p=0.02). There was no difference in rates of complications in patients with free or pedicled flaps, tumors < or > 5cm, distal or proximal location of tumour. The rates of negative excision margins (80%) and wound complications in patients who required reconstructive flaps were not different from that for the other patients treated at our center who did not require reconstructive flaps.
*Conclusion:* The use of soft tissue reconstructive flaps did not reduce the risk of positive excision margins or the rates of wound complications. Pre-operative radiotherapy and tumors located in the lower limb are the main risk factors for wound complications after the use of reconstructive flaps. The risk of amputation secondary to flap complication or failure is low.

**108 The Indications and Prognostic Significance of Amputation for Soft Tissue Sarcoma of the Extremity**

A T Abudu[1], N Driver[1], J S Wunder[1], A M Griffin[1], D Pearce[1], B O O'Sullivan[2], C N Catton[2], R S Bell[1], A M Davis[1]
*[1]University Musculoskeletal Oncology Unit, Mount Sinai Hospital, University of Toronto, [2]Department of Radiation Oncology, Princess Margaret Hospital*

*Objective:* Limb preserving surgery has become the treatment of choice for patients with soft tissue sarcoma but a small group of patients remain unsuitable for limb preserving surgery and require amputation for the local control of their sarcoma. The prognostic significance of the need for amputation to achieve local control in patients with soft tissue sarcoma is unclear. We studied patients with soft tissue sarcoma of the extremity to determine the reasons for amputation, and the outcome following amputation compared to those patients treated with limb preserving surgery.
*Methods:* The clinical charts, prospectively collected database records, radiological investigations and pathological records of patients with soft tissue sarcoma treated at our centre were studied. The patients who had primary amputation, and those who had amputation after complications of limb sparing surgery, were studied to identify the reasons for amputations.
*Results:* 812 consecutive patients were studied. 774 patients (95.3%) had limb-sparing surgery and 38 (4.7%) had primary amputations. Patients with primary amputations were statistically more likely to have metastases at presentation (16% versus 7%), high grade tumours (82% versus 49%), larger tumours (median diameter 11cm versus 7cm) and were older (61 versus 53 years). Multivariate analysis using time dependent Cox model in patients with localised disease at diagnosis revealed that the requirement for primary amputation was a poor prognostic factor for overall and disease free survival independent of tumour grade, tumour size and patients' age. The reasons for primary amputation were: 1) tumour excision would result in inadequate function (13 of 38 patients); 2) large extracompartmental tumours with composite tissue involvement including major vessels, nerves and bone (eight of 38 patients); 3) local recurrence in previously irradiated field (seven of 38 patients); 4) involvement of major neurovascular structures (five of 38 patients); 5) prior unplanned resection with extensive tissue contamination (three of 38 patients); 6) multifocal disease (one of 38 patients) and 7) concurrent peripheral vascular disease (one of 38 patients).
*Conclusion:* Patients who require primary amputations are a select group with unfavourable tumour characteristics and have a poor prognosis. The are likely to present with metastases and those with localised disease at the time of treatment develop metastases much earlier compared to patients in whom limb preserving surgery is possible. The requirement for a primary amputation is an independent poor prognostic factor in patients with localised disease at diagnosis.

**110 Low Dose Radiotherapy and Staged Surgical Resection For Diffuse Pigmented Villonodular Synovitis of the Knee Preserves Normal Knee Function with Minimal Risk of Recurrence**

Marc Rankin[1], Felasfa M. Wodajo[2], James Wittig[2], Kari Mansour[2], Martin M. Malawer[2]
*[1]Division of Orthopedics, Howard University Hospital, [2]Washington Cancer Institute, Washington Hospital Center*

*Objective:* Pigmented villonodular synovitis (PVNS) of the knee is a benign, proliferative synovial disorder characterized by knee pain and recurrent effusions. Recent karyotype chromosomal analysis suggest that it is a true neoplasm and not an inflammatory process. There is a localized and a diffuse form of the disorder with the localized or nodular version amenable to limited, even arthroscopic resection. The treatment strategy for diffuse PVNS, however, has to address the tendency of the lesion to recur quickly following an incomplete resection. Our objective is to evaluate the results of a uniformly applied, multimodal treatment protocol for diffuse PVNS consisting of surgery and radiation.
*Methods:* During the period of 1990 to the present, 19 patients underwent surgical resection for PVNS of the knee by the same surgical team. Of these, 8 patients underwent staged procedures for diffuse PVNS consisting of an anterior arthrotomy and synovectomy followed 3 months later by posterior arthrotomy and synovectomy. After wound healing was complete, patients underwent external beam radiotherapy, most commonly consisting of 3000 cGy in 15 fractions. Patients were followed postoperatively at 3–6 month intervals by physical exam and magnetic resonance imaging (MRI). Five patients who completed treatment between 1995 and 1998 form the basis for this report.
*Results:* The study group consists of three females and two males with an average age of 25 years (range 14–43 years) at the time of surgery. The average follow-up interval was 30 months (range 5–57 months). Three patients presented with recurrent disease after either an anterior arthrotomy or arthroscopy by another surgeon, one patient presented after two previous arthroscopies. Average post-operative knee extension at the most recent examination was +3 deg (range 0–15 deg) and average flexion was 127 deg (range 115–135 deg). Four of five patients had no complaints of pain nor reported any restriction of desired activities; the youngest two patients (17 and 25 years old) were athletically active. Four patients had no knee effusion at their last visit. One patient who was 47 years old had a small effusion and occasional activity related pain and was noted radiographically to have developed a moderate amount of degenerative osteoarthrosis of the knee in the five year interval since treatment. There were no recurrences.
*Conclusion:* As evidenced by the finding that 4 out of 5 of the study patients presented to us with recurrent disease, anterior synovectomy or arthroscopy alone is probably inadequate treatment for this disorder. The extent of tumor in diffuse PVNS is easily underestimated and requires thorough anterior and posterior knee exploration as tumor often extends extraarticularly, particularly posteriorly along the semimembranous tendon complex. Most published reports on knee PVNS are generally retrospective analyses, often incorporating various treatments and extents of disease. We have demonstrated that a protocol consisting of staged anterior and posterior synovectomy with adjuvant low dose external beam radiation for diffuse PVNS of the knee can provide excellent functional results with a low risk of recurrence.

**121 Intraperitoneal Chemotherapy (IPC) After Complete Resection of Peritoneal Sarcomatosis (PS): Results of a Monocentric Randomized Study**

Bonvalot Sylvie, Calvacanti Andrea, Elias Dominique, Terrier Philippe, Vanel Daniel, Lepechoux Cecile, Le Cesne Axel
*Institut Gustave Roussy*

*Objective:* In order to decrease locoregional relapse after complete resection of PS, the role of IPC was prospectively evaluated.
*Methods:* Patients (pts) with complete resection of PS were randomized between adjunction of IPC or not. IPC consisted of Doxorubicin, 0.1 mg/kg and Cisplatin, 15 mg/m2 every day for 5 consecutive days.
*Results:* Thirty-eight consecutive pts have been enrolled in the study, 19 in each group (IPC-, IPC+) with a M/F sex ratio of 14/24. Median age was 58 (39 to 72) and 48 yrs (31 to 71) in IPC- and IPC+ group respectively. Ratio of retroperitoneal (RPS) and visceral (VS) sarcomas were 9/10 and 6/13 in IPC- and IPC+ group respectively. Histoprognostic grade and median number of organs resected during surgery were similar in both groups. Sugarbaker score of sarcomatosis were 13 (3, 27) and 13,7 (2, 20) in IPC- and IPC+ respectively. There was no toxic deaths and morbidity was similar in both groups (4 pts in each group). Median time of hospitalization was 22 days (range 11 to 39) for IPC- and 24 days (range 15 to 42) for IPC+. The median follow-up is 36 months. The median local relapse-free survival and overall survival were identical in both groups, 12.5 and 18 months respectively with no difference between RPS and VS.
*Conclusion:* Addition of IPC did not modify outcome of pts after complete resection of RPS and VS. OS and DFS of this study are similar to those observed in phase II studies combining IPC with hyperthermia. An optimal surgery of PS remains the only prognostic factor for survival.

**128 Is there a Role for Isolated Limb Perfusion in Unresectable Extremity Sarcomas?**

Barry W. Feig
*The University of Texas M.D. Anderson Cancer Center*

*Objective:* A number of recent studies from Europe have reported on the efficacy of isolated limb perfusion (ILP) in patients with unresectable extremity sarcomas. Unfortunately, these results have been difficult to reproduce in studies performed in the U.S. As systemically delivered doxorubicin has shown the most activity in patients with soft tissue sarcomas, we were interested in evaluating the use of doxorubicin delivered via ILP. We therefore undertook a Phase I trial of ILP with doxorubicin using the dosing previously reported from a phase I dose escalation trial from Italy. (Rossi et al. Cancer 73:2140-2146, 1999)
*Methods:* Seven patients have been entered in this Phase I trial; 6 patients have been perfused, the seventh patient was unable to be perfused secondary to inadequate venous access. There were 3 males and 3 females with a median age of 33 (range 19–75). The initial 3 patients were treated at a dose of 1.4 mg/kg for the lower extremity (LE) and 0.7 mg/kg for the upper extremity (UE). The dose was lowered 20% in the second 3 patients to 1.0mg/kg and 0.5mg/kg for the LE and UE respectively, secondary to toxicity seen in the first 3 patients. The perfusion was performed using a bubble oxygenator, roller pump in the standard, previously described manner. The perfusion with doxorubicin was performed for 60 minutes at normothermic temperatures (37oC). Patients were then evaluated for response to treatment using MRI scan at 6 weeks post perfusion. At that time patients underwent marginal excisions of the tumors.
*Results:* In the 6 patients thus far perfused, there have been no responses seen. Three patients had evidence of disease progression at the time of their 6 week MRI evaluation. There was one grade 3, and one grade 2 neurologic toxicity. Two patients have undergone amputation secondary to progression of disease. Two patients have had evidence of significant myonecrosis based on elevated CPK levels post-operatively (27,000 and 31,000) with significant post-operative limb dysfunction requiring extending physical therapy treatments.
*Conclusion:* Thus far, there have been no responses observed in this study population. Additionally, there have been significant toxicities observed, as described above in the results section. The lack of responses seen to ILP with doxorubicin in this study, as well as the low response rates seen in other perfusion studies performed in the United States, makes it unlikely that there is a role for ILP in patients with unresectable extremity sarcomas.

**132 Phase I Trial of Preoperative Doxorubicin-Based Concurrent Chemoradiation and Surgical Resection For Localized, Resectable Extremity and Trunk Soft Tissue Sarcomas (STS)**

Peter W.T. Pisters, Shreyaskumar R. Patel, Valerae O. Lewis, Kristin L. Weber, Patrick P. Lin, Rex Marco, Alan W. Yasko, Kelly K. Hunt, Barry W. Feig, Jonathan C. Trent II
*The University of Texas M. D. Anderson Cancer Center*

*Objective:* Doxorubicin is active against soft tissue sarcoma and is a potent radiosensitizer. However, no prior studies have examined continuous infusion doxorubicin combined with concurrent radiation for patients with STS. This phase I trial is designed to define the toxicities of concurrent doxorubicin and external beam radiotherapy (EBRT) followed by surgical resection in order to establish the maximum tolerated dose (MTD) of doxorubicin when combined with standard preoperative EBRT.
*Methods:* Patients with localized, resectable grade II or III primary or recurrent STS of the trunk and extremity are eligible. EBRT (2 Gy/fraction, 25 fractions) is provided with continuous infusion doxorubicin (starting dose 10 mg/m$^2$/week, Monday to Thursday, x 5 weeks). Doxorubicin dose escalation is determined by the continuous reassessment method (CRM). 23 patients have been treated. The dose escalation scheme (and number of patients treated) for successive cohorts of patients according to the CRM method has been: 12.5 mg/m$^2$ per week (1 patient), 15.0 mg/m$^2$ per week (3 patients), 17.5 mg/m$^2$ per week (19 patients).

*Results:* The median tumor size was 9.8 cm (range, 1.8–22 cm). Histologies included unclassified sarcoma (8 patients), malignant fibrous histiocytoma (7 patients), liposarcoma (4 patients), and 3 patients with other STS. With 23 of 30 planned patients treated, the MTD by CRM appears to be 17.5 mg/m$^2$ per week; 7 patients remain to be treated for complete assessment. Grade III local cutaneous toxicity (confluent moist desquamation) has been observed in 5 patients treated at the 17.5 mg/m$^2$ per week dose level. Grade III neutropenia was observed in 2 patients treated at the 17.5 mg/m$^2$ dose level; no patients have experienced febrile neutropenia.

*Conclusion:* Concurrent continuous infusion doxorubicin-based chemoradiotherapy appears feasible and safe. The MTD of doxorubicin appears to be 17.5 mg/m$^2$ per week when doxorubicin is administered by continuous infusion over 4 days/week for 5 successive weeks with 50 Gy of preoperative EBRT.
